# Emergence of *MUC1* in Mammals for Adaptation of Barrier Epithelia

**DOI:** 10.3390/cancers14194805

**Published:** 2022-09-30

**Authors:** Donald W. Kufe

**Affiliations:** Dana-Farber Cancer Institute, Harvard Medical School, 450 Brookline Avenue, D830, Boston, MA 02215, USA; donald_kufe@dfci.harvard.edu

**Keywords:** MUC1, MUC1-C, barrier epithelia, loss of homeostasis, inflammatory memory, cancer

## Abstract

**Simple Summary:**

The evidence reviewed here indicates that MUC1-C evolved in mammals to promote inflammatory adaptation in barrier tissues that extends to resident stem cells and immune cells. Inflammatory memory is an essential process that protects barrier niches against future insults. Evolutionary adaptations arising from natural selection, as for example the *MUC1* gene, can be beneficial for survival. However, prolonged activation of MUC1-C in settings of chronic inflammation represents an adverse adaptation that promotes cancer.

**Abstract:**

The *mucin 1* (*MUC1*) gene was discovered based on its overexpression in human breast cancers. Subsequent work demonstrated that MUC1 is aberrantly expressed in cancers originating from other diverse organs, including skin and immune cells. These findings supported a role for MUC1 in the adaptation of barrier tissues to infection and environmental stress. Of fundamental importance for this evolutionary adaptation was inclusion of a SEA domain, which catalyzes autoproteolysis of the MUC1 protein and formation of a non-covalent heterodimeric complex. The resulting MUC1 heterodimer is poised at the apical cell membrane to respond to loss of homeostasis. Disruption of the complex releases the MUC1 N-terminal (MUC1-N) subunit into a protective mucous gel. Conversely, the transmembrane C-terminal (MUC1-C) subunit activates a program of lineage plasticity, epigenetic reprogramming and repair. This MUC1-C-activated program apparently evolved for barrier tissues to mount self-regulating proliferative, inflammatory and remodeling responses associated with wound healing. Emerging evidence indicates that MUC1-C underpins inflammatory adaptation of tissue stem cells and immune cells in the barrier niche. This review focuses on how prolonged activation of MUC1-C by chronic inflammation in these niches promotes the cancer stem cell (CSC) state by establishing auto-inductive nodes that drive self-renewal and tumorigenicity.

## 1. Introduction

### 1.1. Discovery of MUC1 in Breast Cancer

The DF3 high molecular weight antigen was identified in human breast cancers [1]. DF3 expression was found to be upregulated in the cytoplasm and over the entire surface of breast cancer cells. In contrast, DF3 was expressed at lower levels and restricted to the apical membranes of normal mammary epithelial cells [1]. Studies of human milk fat globules identified a high molecular weight polymorphic epithelial membrane antigen (EMA/PEM) [2,3,4]. The expression of EMA/PEM was similarly upregulated in human breast cancers and aberrantly distributed over the cell membrane [3,4]. Further characterization of DF3 and EMA/PEM demonstrated the same unique structures of *O*-glycosylated 20 amino acid (aa) tandem repeats [5,6,7,8]. Based on these and other findings, DF3 and EMA/PEM were designated as the founding member, or mucin 1 (MUC1), of a family of 21 genetically distinct secreted and transmembrane mucins [9,10,11,12].

The early work on aberrant MUC1 expression in breast cancer was extended by studies in other types of adenocarcinomas and squamous cell carcinomas, as well as hematologic malignancies, that continue to shed light on the involvement of MUC1 in cancer initiation and progression. These studies, which include meta-analyses, have largely demonstrated that MUC1 is upregulated in diverse cancers and is associated with poor patient outcomes [13]. Notably, however, interpretation of these findings has often been confounded by the criteria used to assess MUC1 expression; that is, for example, membrane vs. cytoplasmic localization. Other criteria using MUC1 expression at the mRNA vs. protein levels can lead to conflicting results in that MUC1 expression is regulated by multiple transcriptional and posttranscriptional mechanisms. Moreover, MUC1 encodes two subunits. The evidence indicates that the transmembrane MUC1 subunit, which localizes to the cytoplasm and nucleus, is of importance for driving cancer progression and the cancer stem cell (CSC) state [14,15,16]. Studies assessing expression of this subunit may therefore provide clarity to the field.

### 1.2. Evolution of MUC1 in Mammals

The *MUC1* gene first appeared in mammals [17]. Many of the genes that arose in early mammalian evolution encode proteins expressed in the mammary gland, skin and immune cells [18]. Other early mammalian genes are preferentially activated in testes, supporting the potential for sexual selection.

In mammals, barrier epithelia form the interface with the external environment [19]. MUC1 is expressed in barrier epithelia lining the (i) gastrointestinal, respiratory and genitourinary tracts and (ii) ducts in specialized organs, such as the liver and pancreas [9]. MUC1 is also expressed in skin, immune cells and reproductive organs, including placenta, ovary and testis [9,20]. These findings have indicated that MUC1 evolved in mammals to play pleotropic roles in (i) protecting barrier epithelia from biotic and abiotic insults arising from exposure to the external environment and (ii) promoting reproductive capacity essential for species propagation.

**Figure 1:**
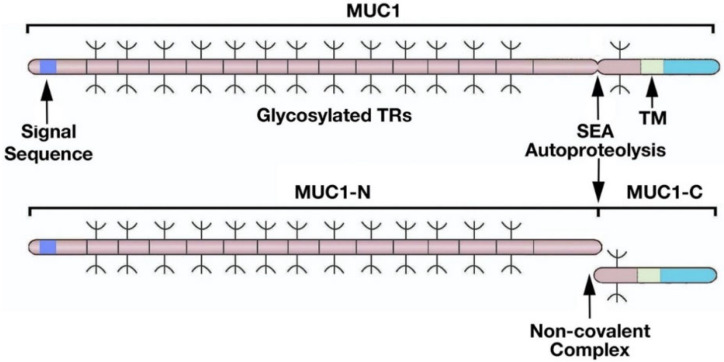
Structure of the MUC1 heterodimeric complex. MUC1 is translated as a single polypeptide that includes a signal sequence for membrane localization and an extended region of O-glycosylated 20 aa tandem repeats (TRs). MUC1 also includes a SEA domain and a transmembrane (TM) domain. Autoproteolysis within the SEA domain results in the generation of MUC1 N-terminal (MUC1-N) and C-terminal (MUC1-C) subunits that form a non-covalent complex. Figure modified from Kufe [9].

Certain of the new mammalian protein-encoding genes emerged de novo from noncoding genomic regions [18]. *MUC1* has no homology with other genes except for sequences encoding a ~122 aa sea urchin sperm protein-enterokinase-agrin (SEA) domain [17,21,22,23]. Ancient evolutionary origin of the SEA domain has played roles in the autoproteolysis of cell surface and secreted proteins [23]. Localization of SEA domains adjacent to a transmembrane region occurs in type I cell surface proteins that, in addition to MUC1, include dystroglycan and NOTCH [9,23]. SEA domains in mammalian genes have diversified with functions of the gene products. The MUC1 SEA domain includes a 68 aa conserved region and an additional 54 aa that dictate MUC1 autoproteolysis and, in turn, the formation of a heterodimeric complex (Figure 1) [14,17].

**Figure 2:**
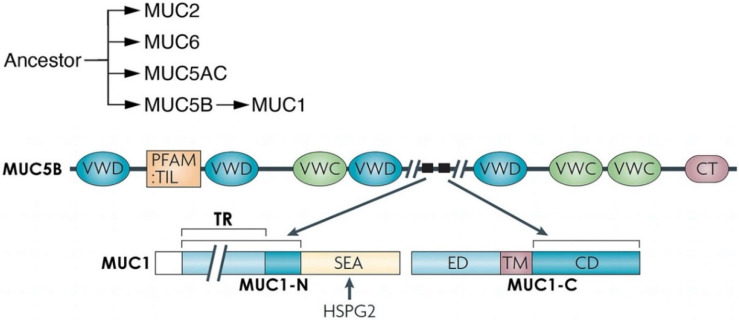
MUC1 evolved from MUC5B and HSPG2. The secreted MUC2, MUC6, MUC5AC and MUC5B mucins, which localize to chromosome 11p15.5 in humans, emerged from common ancestors that appeared in early metazoan evolution. MUC1 emerged in mammals in part from MUC5B and is located at chromosome 1q22. The MUC1 SEA domain evolved from HSPG2. MUC5AC and MUC5B form protective oligomeric structures in the response to inflammation [29]. MUC1 is devoid of the von Willebrand factor type C (VWC) and D (VWD), trypsin inhibitor-like cysteine-rich (TIL) and C-terminal cysteine knot (CT) domains. HSPG2/perlecan is activated by inflammation and has been linked to regulation of the tumor microenvironment and cancer progression [30]. Figure modified from Kufe [9].

Autoproteolysis of MUC1 between glycine and serine at a GSVVV motif in the SEA domain generates N-terminal (MUC1-N) and C-terminal (MUC1-C) subunits (Figure 1) [9,14]. In turn, MUC1-N and MUC1-C form a non-covalent complex mediated by a leucine zipper-like structure (Figure 1) [24]. The MUC1-N and MUC1-C subunits are distinct proteins and are designated differently than isoforms, which are commonly identified by Greek letters (i.e., α, β, γ). The MUC1-N subunit consists of a signal-peptide sequence for cell membrane localization [9,14]. MUC1-N also includes tandem repeats (TRs) that are highly glycosylated and are a common physical, but genetically distinct, characteristic of the family of secreted and transmembrane mucins. The region of MUC1-N between the TRs and SEA domain emerged from sequences in the MUC5B secreted mucin (Figure 2) [17]. MUC1-C also evolved in part from MUC5B (Figure 2) [17]. The MUC5B locus is located on chromosome 11p15.5 with genes encoding the MUC2, MUC6 and MUC5AC secreted mucins that appeared in early metazoan evolution for forming protective gels [25,26,27]. Of these mucins, MUC5B is required for mucociliary clearance and immune homeostasis in the respiratory tract [28].

## 2. Importance of MUC1 Structure for Barrier Tissue Function

Simple epithelial barriers lining internal mammalian organs consist of a single layer of polarized cells. The skin epidermis evolved with multiple overlying layers of squamous cells that are separated by lipid bilayers [31]. Both barriers afford physical protection and secrete defensin antimicrobial peptides [32]. These barriers are also dependent on stem cells for regeneration in response to loss of integrity from damage or infection [32]. MUC1 contributes to physical protection of barrier tissues through the MUC1-N subunit as an integral component of the cell surface glycocalyx and mucous gel [9,14]. Moreover, the MUC1-C subunit has the capacity for activating stem cell functions in repair and remodeling for regeneration of the barrier [14].

**Figure 3:**
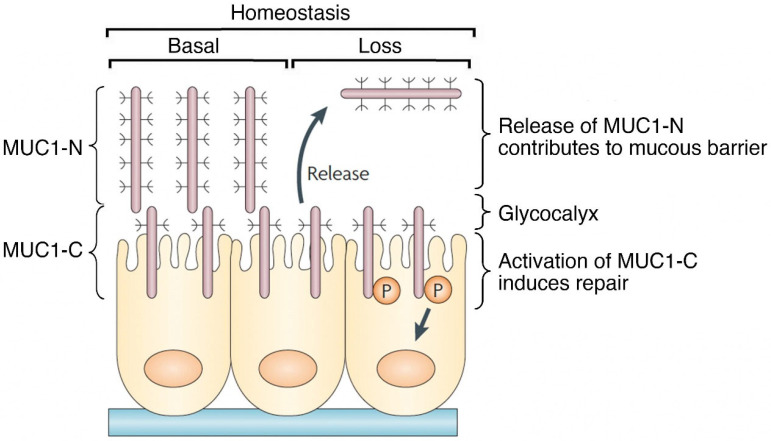
MUC1 contributes to a protective physical barrier and activation of repair in epithelia. The MUC1 heterodimer is poised in a basal state at the apical cell membrane as a sensor of homeostasis. Entropic forces in the glycocalyx induced in association with loss of homeostasis, as well as proteolytic cleavage, disrupt the MUC1-N/MUC1-C complex with release of MUC1-N into a protective physical barrier. Activation of MUC1-C induces a program of repair associated with the wound healing response. Figure modified from Kufe [9].

The diverse functions of MUC1 in physical protection and stem cell activation of barrier epithelia can, in part, be attributed to the SEA domain. The MUC1 SEA domain is unique in that it generates the MUC1-N and MUC1-C subunits by autoproteolysis and, in turn, their formation of a non-covalent complex [9,14,33]. The signal sequence of the MUC1-N subunit directs the complex to the cell membrane, where MUC1-C positions the heterodimer for activation by loss of homeostasis (Figure 3) [9,14]. The MUC1-N/MUC1-C complex functions by integrating communication between MUC1-N in the glycocalyx and MUC1-C in the cell membrane. In this way, the interaction between MUC1-N and MUC1-C acts as a sensor of entropic forces within the extracellular matrix [34]. Mechanical forces induced by loss of homeostasis disrupt the noncovalent MUC1-N/MUC1-C heterodimer (Figure 3) [35,36]. In addition, proteolytic cleavages of the MUC1-N/MUC1-C heterodimer by MT1-MMP, ADAM17 and gamma-secretase represent other mechanisms for disruption of the complex [37,38,39]. As one consequence, release of MUC1-N into the glycocalyx contributes to the barrier and entraps pathogens for mucociliary excretion (Figure 3) [9,14]. Release of MUC1-N also enables activation of MUC1-C for inducing the epithelial-mesenchymal transition (EMT), repair and potentially reestablishment of homeostasis upon resolution of inflammation (Figure 3).

## 3. MUC1 Responds to Loss of Homeostasis by Inducing Loss of Polarity and EMT

Tissue-specific stem cells require the capability to rapidly respond to loss of barrier integrity by damage and infections. The MUC1-N/MUC1-C complex is poised at the epithelial apical cell membrane as a sensor of changes in the glycocalyx [9,14]. MUC1-N and MUC1-C interact by a non-covalent leucine zipper-like structure with the potential for disruption by entropic forces [24,40]. The associated release of MUC1-N and activation of MUC1-C induce pleotropic events that can contribute to loss of polarity [9,14]. Apical-basal polarity is conferred in part by the Crumbs (CRB) complex. Activation of MUC1-C represses CRB3 with disruption of CRB function [41]. MUC1-C also represses E-cadherin, which is essential for maintenance of polarity by the adherens junction [9,14]. In addition, MUC1-C induces EMT by activating expression of the ZEB1, TWIST1 and SNAIL EMT TFs [42,43]. The findings that MUC1-C drives lineage plasticity, which is an integral component of the wound healing response, provided support for involvement in adaptation of barrier tissues to infection and environmental stress [14].

**Figure 4:**
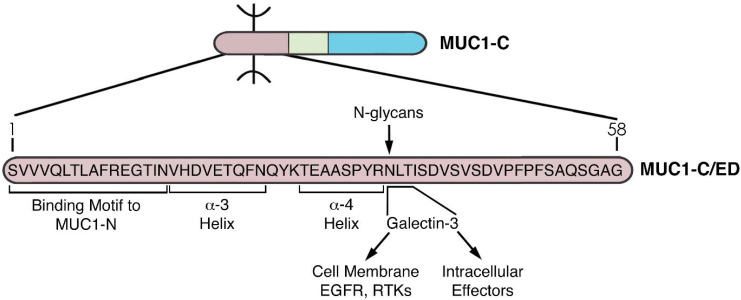
Structure of the MUC1-C extracellular domain (ED). The 58 aa MUC1-C/ED includes the SVVVQLTLAFREGTIN sequence that forms a non-covalent interaction with MUC1-N [24]. Downstream to that region is the VHDVETQFNQ sequence which forms the alpha-3 helix. A QYK motif separates the alpha-3 and alpha-4 helices. Adjacent to the alpha-4 helix is a consensus NLT motif that is modified by N-glycosylation and functions as a galectin-3 ligand binding site. Galectin-3 acts as a bridge for the association of MUC1-C with EGFR and other RTKs at the cell membrane, as well as additional effectors in the cytoplasm and nucleus.

MUC1-C has a unique structure that is highly conserved across mammals [14]. The MUC1-C extracellular domain (ED) is 58 aa in length and plays important roles in MUC1-C function. MUC1-C/ED aa at positions 1–16 confer association with MUC1-N (Figure 4). MUC1-C/ED includes alpha-3 (aa 17–25) and alpha-4 (aa 28–35) helices (Figure 4), which likely contribute to the capacity of full-length MUC1-C, particularly the cytoplasmic domain, to interact with diverse intracellular effectors. MUC1-C/ED also includes a consensus NLT motif (aa 36–38) that is modified by N-glycans [44]. The NLT site is adjacent to the alpha-4 helix and thereby could be affected as a substrate for N-glycosylation by the alpha-4 helical structure (Figure 4). In this regard, MUC1-C is expressed as N-glycosylated (20–25 kDa) and unglycosylated (17 kDa) forms [44]. This distinction in MUC1-C 20–25 and 17 kDa species is of potential functional significance. The MUC1-C N-glycosylated NLT site is a substrate for galectin-3 binding [44]. In turn, galectin-3 acts as a bridge for MUC1-C interactions with cell surface receptors, such as EGFR, as well as diverse intracellular effectors, including the nuclear ribonucleoprotein complex and calcium channel TRPV5 (Figure 4) [44,45,46,47]. Conversely, the unglycosylated MUC1-C 17 kDa form lacks this capacity to interact with intracellular effectors by galectin-3-mediated mechanisms.

## 4. MUC1-C Functions as a Node for Activation of the Proliferative WNT/β-Catenin Pathway

**Figure 5:**
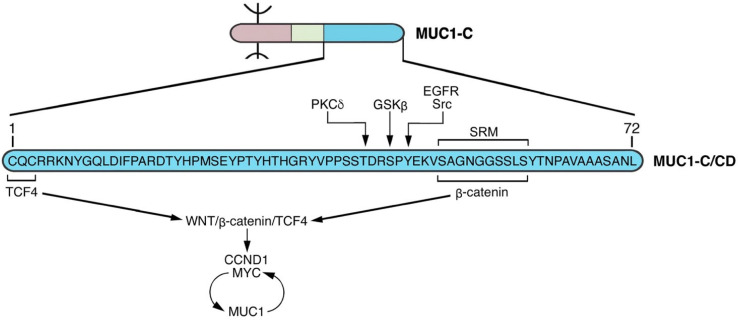
The MUC1-C cytoplasmic domain (CD) functions as a node (NODE 1) in auto-induction of the WNT/β-catenin signaling pathway. The 72 aa MUC1-C/CD includes a serine-rich motif (aa 50–59, SAGNGGSSLS; SRM) that interacts directly with the β-catenin Armadillo repeats [60]. This interaction contributes to stabilization of β-catenin and is regulated by upstream phosphorylation of (i) T-41 by PKCδ [59], (ii) S-44 by GSK3β [60], and (iii) Y-46 by EGFR and SRC [57,58]. The MUC1-C/CD CQC motif is necessary for interactions with certain proteins that are mediated by disulfide bonds [48]. In this way, the MUC1-C/CD CQC motif binds to the TCF4 E-tail [61], facilitating the formation of β-catenin/TCF4 complexes that activate CCND1 and MYC. MUC1-C forms auto-inductive circuits with β-catenin/TCF4 and MYC in sustaining activation of this node. Notably, the CQC motif Cys residues are subject to palmitoylation [50], which could preclude their capacity to form disulfide bonds and function in this and other auto-inductive nodes.

The MUC1-C cytoplasmic domain is a 72 aa intrinsically disordered protein devoid of enzymatic function (Figure 5). For clarity here, we use the nomenclature aa 1–72 in that the MUC1-C/ED can be subject to proteolytic cleavage at different sites, affecting the downstream numbering of the cytoplasmic domain residues.

MUC1-C/CD includes a CQC motif (aa 1–3) that is activated by loss of homeostasis and increases in reactive oxygen species (ROS) [48,49]. Activation of the CQC motif is necessary for MUC1-C homodimerization and heterodimeric interactions with certain other proteins [14,48,49]. MUC1-C homodimers form complexes with RTKs, such as EGFR, at the cell membrane and contribute to transduction of their downstream signaling pathways [9,14]. Cell membrane-associated MUC1-C is subject to palmitoylation and trafficking to endosomes [50,51]. There, MUC1-C is transported by HSP70/HSP90 to the mitochondrial outer membrane, where it inhibits the intrinsic apoptotic pathway [52,53,54]. MUC1-C is also imported into the nucleus by interactions with the nuclear pore complex [14,48]. These effects of loss of homeostasis and disruption of redox balance on intracellular localization of MUC1-C are in principle reversible; however, they have the potential for being established in settings of chronic inflammation and cancer progression.

The MUC1-C/CD also includes a serine-rich motif (SRM; aa 50–59) with similarity to β-catenin binding regions found in E-cadherin, adenomatous polyposis coli (APC) and other proteins (Figure 5). The finding that the MUC1-C/CD SRM interacts directly with β-catenin provided early evidence for a role in intracellular signaling [55]. Subsequent studies demonstrated that phosphorylation of MUC1-C/CD by GSK3β, SRC, EGFR and PKCγ at sites just upstream of the SRM contribute to stabilization of β-catenin and activation of the WNT/β-catenin pathway (Figure 5) [55,56,57,58,59,60]. These findings are highlighted in that they support the recurring theme that MUC1-C acts as a self-regulating node in integrating multiple signals; that is, in this case (NODE 1), phosphorylation of an intrinsically disordered region that activates the WNT/β-catenin pathway (Figure 5).

Binding of the MUC1-C SRM to β-catenin could have an allosteric effect on other MUC1-C/CD regions, as is often found in intrinsically disordered proteins [62]. β-catenin-mediated gene transcription is dependent in large part on interactions with the TCF4/TCF7L2 TF [61]. MUC1-C facilitates formation of β-catenin/TCF4 heterodimers by binding directly to both the β-catenin Armadillo repeats and to the TCF4 E-tail (Figure 5) [60,61]. MUC1-C/β-catenin/TCF4 complexes occupy the *CCND1* promoter and activate its transcription by recruiting p300 and increasing H3K27 acetylation [61]. A similar mechanism extends to MUC1-C activation of the MYC gene; that is, occupancy of the *MYC* promoter by MUC1-C/β-catenin/TCF4 complexes and activating transcription by the H3K27ac modification [63]. In the setting of MUC1-C-induced *MYC* activation, MUC1-C forms a complex with the MYC HLH-LZ region that is of importance for interactions with MAX and the MYC transactivation function [64].

MUC1-C activates MYC target genes that encode BMI1/PRC1 and the NuRD chromatin remodeling complex [64]. MUC1-C also activates the MYC pathway in association with induction of E2F target genes and dysregulation of mitotic progression [65]. These findings and those demonstrating that MUC1-C→E2F1 signaling activates the SWI/SNF BAF and PBAF chromatin remodeling complexes provided support for involvement of MUC1-C in coupling proliferative responses with the regulation of chromatin architecture [66,67,68]. 

The interaction between MUC1-C and MYC exemplifies a theme for MUC1-C signaling; that is, activation of a gene and interaction with the product of the same gene. Other selected examples include, TAK1 [69], ZEB1 [42], TWIST1 [43], EZH2 [70] and BMI1 [71]. In this way, MUC1-C can rapidly intersect with certain signaling proteins and then sustain or amplify that interaction by inducing expression of the gene encoding that effector. 

## 5. MUC1-C Acts as a Node for Promoting Chronic Inflammation

**Figure 6:**
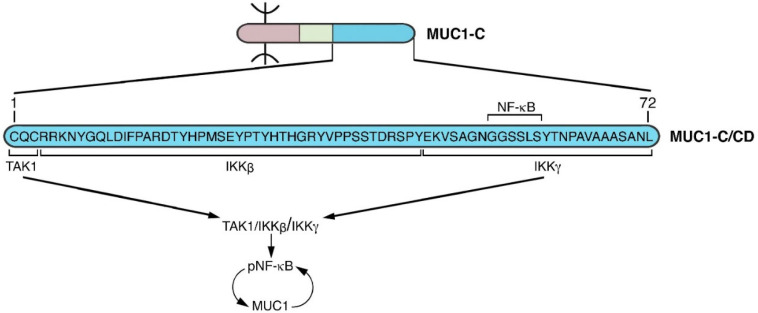
MUC1-C/CD functions as a node (NODE 2) for auto-induction of the TAK1/IKK/NF-κB pathway. The MUC1-C/CD CQC motif binds to TAK1 [69]. MUC1-C/CD(4–45) interacts with IKKβ and MUC1-C(46–72) forms a complex with IKKγ [81]. The MUC1-C/CD SRM GGSSLS sequence binds directly to the NF-κB p65 RHD/DBD, integrating activation of the TAK1/IKK/NF-κB pathway [77]. NF-κB induces MUC1 expression in an auto-inductive circuit.

WNT signaling is involved in multiple processes that include proliferation, wound healing and cancer progression [72,73]. The wound healing response is orchestrated in part by integration of proliferation and inflammation [74,75,76]. In parallel with induction of WNT and E2F signaling, MUC1-C activates the proinflammatory TAK1→IKK→NF-κB p65 pathway (Figure 6) [69,77,78]. The MUC1-C SRM binds to the NF-κB Rel Homology Domain (RHD), which includes the DNA binding domain (DBD) and promotes the transcription of NF-κB target genes (Figure 6) [77,78]. As one example, MUC1-C→NF-κB signaling activates the *ZEB1* gene and, in turn, MUC1-C associates with ZEB1 in driving EMT [42]. MUC1-C/ZEB1 complexes also suppress CRB3 and E-cadherin in coupling loss of polarity with EMT [78]. Other downstream effectors of the MUC1-C→NF-κB pathway encompass induction of the (i) DNA methyltransferases (DNMTs) with repression of TSGs [79], (ii) BMI1-stemness factor and component of the Polycomb Repressive Complex 1 (PRC1) [71], and (iii) PD-L1 mediator of immune evasion [80]. Of significance, MUC1-C→NF-κB signaling also induces the *MUC1* gene in an auto-inductive circuit [77]. In this way, MUC1-C functions as a node (NODE 2) in activating the inflammatory NF-κB pathway with induction of EMT, epigenetic reprogramming and stemness (Figure 6).

**Figure 7:**
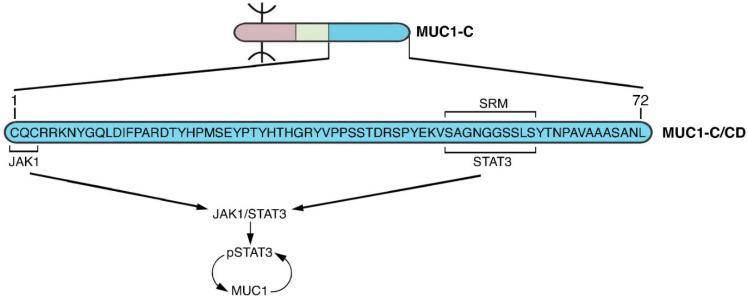
MUC1-C activates JAK1→STAT3 signaling in an auto-inductive loop (NODE 3). As found for TAK1 in the NF-κB pathway, the MUC1-C/CD CQC motif binds to JAK1. In addition, like NF-κB p65, the MUC1-C/CD SRM functions as a site for direct binding to the STAT3 DBD and for JAK1-mediated pSTAT3 activation [82]. In turn, pSTAT3 induces MUC1 expression in another auto-inductive inflammatory circuit that parallels the node driving MUC1-C→NF-κB signaling.

Interaction of MUC1-C with STAT3 complements the MUC1-C→NF-κB pathway in fostering chronic inflammation. MUC1-C forms complexes with JAK1 and STAT3 and induces JAK1-mediated STAT3 phosphorylation (Figure 7) [82]. As a result, MUC1-C contributes to activation of STAT3 by IL-6, IL-10 and IL-22 [82]. Binding of MUC1-C to the STAT3 DBD induces expression of the *CCND1* target gene [82]. MUC1-C/STAT3 complexes also activate the *TWIST1* gene and thereby the induction of ZEB1, SNAIL and EMT [43]. As found for NF-κB, the MUC1-C→STAT3→TWIST1 pathway couples EMT with induction of BMI1, as well as the SOX2, ALDH1 and CD44 stem cell markers [43]. In addition, and like NF-κB, MUC1-C/STAT3 complexes activate the *MUC1* promoter in another cell autonomous, self-regulating circuit (Figure 7) [82]. MUC1-C thereby functions as a distinct node for activating STAT3 and interconnecting inflammation, EMT and stemness (NODE 3) (Figure 7). Prolonged activation of both the MUC1-C→NF-κB and MUC1-C→STAT3 pathways could also intersect by acting in concert and promoting a state of chronic inflammation.

## 6. MUC1-C and Inflammatory Memory

Epithelial and immune cells coordinate efforts in the response to inflammation [32]. As an integral component of this coordination, these cell populations have the capacity for remembering inflammatory insults [32,83]. Inflammatory memory confers the ability to recall initial insults and respond more robustly to subsequent biotic and abiotic exposures [32,83]. In line with this capacity, adaptation of barrier tissues to infections and environmental stress has been essential for the survival of mammals. Inflammatory memory and adaptation are associated with chromatin remodeling of key genes in this process. Along these lines, activation of the STAT3 pathway contributes to the establishment of inflammatory memory [84]. STAT3 cooperates with the AP-1 family of stress-associated pioneer TFs in maintaining memory [84]. Of potential significance in this regard, MUC1-C activates JUN/AP-1 [68], which plays pleotropic roles in inflammation, proliferation, and wound repair [85,86]. NF-κB also coordinates transcriptional memory responses to inflammatory stimuli [87]. MUC1-C thus interacts with STAT3 and NF-κB in activating their target genes by mechanisms involving epigenetic reprogramming, which could form the basis for establishing inflammatory memory in CSCs.

**Figure 8:**
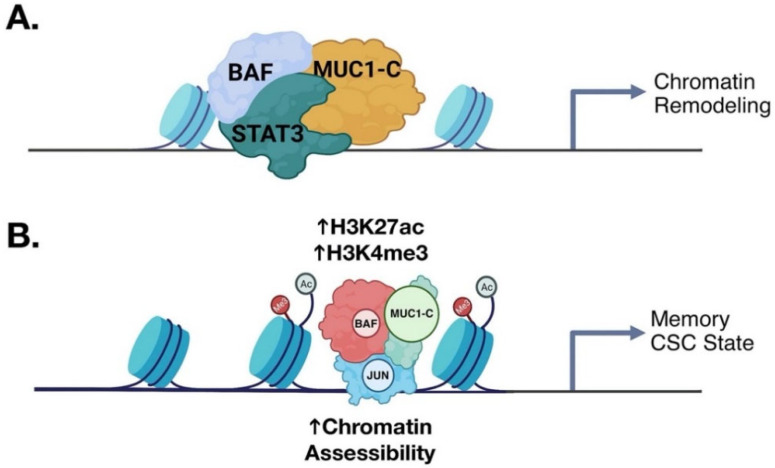
MUC1-C promotes chromatin remodeling in coupling memory with progression of the CSC state. (**A**). The inflammatory MUC1-C→STAT3 auto-inductive node induces the EMT program, stem cell factors and CSC state as evidenced by self-renewal capacity and tumorigenicity [43]. MUC1-C/STAT3 complexes recruit the BAF chromatin remodeling complex to specific target genes to regulate chromatin accessibility (BioRender). (**B**). Proposed model in which MUC1-C and BAF recruit JUN/AP-1 to establish memory and activation of stemness-associated genes in sustaining the CSC state [68]. Modified from Bhattacharya using BioRender [68].

The MUC1→STAT3 pathway induces EMT and stemness by epigenetic reprogramming [15,43]. MUC1-C→NF-κB signaling activates the (i) RING1 member of PRC1, and (ii) EZH2 and SUZ12 components of PRC2 in epigenetic reprogramming and chromatin remodeling [78,88,89]. PRC1/2 driven gene repression is counteracted by the SWI/SNF chromatin remodeling complexes [90,91]. MUC1-C activates the SWI/SNF BAF and PBAF complexes in CSCs, coupling epigenetic reprogramming and chromatin remodeling (Figure 8A) [66,67]. In concert with these interactions, MUC1-C regulates genome-wide accessibility of chromatin (Figure 8A) [68]. In potential concordance with inflammatory memory [84], MUC1-C recruits JUN/AP-1 to gene enhancers with increases in chromatin accessibility (Figure 8B) [68]. In addition, MUC1-C orchestrates chromatin remodeling with induction of the Yamanaka pluripotency factors (OCT4, SOX2, KLF4, MYC) in CSCs [15,92,93,94], which function in reprogramming of the epigenome [95].

These findings provided new insights into a pivotal role for MUC1-C in integrating lineage plasticity and chromatin remodeling, which are transient in wound repair and sustained in promoting the CSC state. 

## 7. MUC1-C Is Necessary for the CSC State

Activation of MUC1-C in chronically inflamed intestinal epithelial cells promotes the progression of colitis to colorectal cancer [93]. MUC1-C is also necessary for progression of castration-resistant prostate cancer (CRPC) to the dedifferentiated form of neuroendocrine prostate cancer (NEPC) [92]. In addition, dependence on MUC1-C has been demonstrated in (i) pancreatic ductal carcinoma with neuroendocrine (NE) dedifferentiation [94], (ii) small-cell lung cancer (SCLC) [65] and (iii) Merkel Cell Carcinoma (MCC) of the skin [96]. MUC1-C is required for the CSC state in these aggressive cancers, as evidenced by dependence on MUC1-C for self-renewal capacity and tumorigenicity [65,92,94,96]. MUC1-C induces lineage plasticity and chromatin remodeling, which are both requisite characteristics of the CSC state [14,15,16]. How MUC1-C precisely contributes to the CSC state is of potential importance for cancer treatment. 

MUC1 auto-inductive loops with STAT3 and NF-κB are of potential importance for establishing maladaptive pathways that, in settings of chronic inflammation, could promote the CSC state. The involvement of MUC1-C in chromatin remodeling has provided additional evidence that changes in chromatin architecture could become irreversibly established by prolonged MUC1-C activation. Chronic inflammation is a widely established driver of cancer initiation and progression, albeit by unclear unifying mechanisms [97]. The importance of MUC1-C as a link between chronic inflammation and cancer has largely remained an unrecognized concept [14,15,16]. Nonetheless, MUC1-C dependence of CSCs across divergent cancers supports a maladaptation that emerged from fundamental processes, such as the wound healing response and inflammatory memory. Along these lines of thinking, immune cells of the lymphoid and myeloid lineages are constituents of epithelial barrier niches [32]. Little is known about the involvement of MUC1-C in inflammatory adaptation of hematopoietic cells. However, MUC1-C is necessary for the CSC state of acute myeloid leukemia [98], cutaneous T cell lymphoma [99] and multiple myeloma [100] cells. These findings invoke the possibility that communication between epithelial-resident stem cells and immune cells in establishing inflammatory memory could extend to MUC1-C dependence in their malignant counterparts.

### Targeting MUC1-C-Driven Auto-Inductive Nodes in CSCs for Cancer Treatment

**Figure 9:**
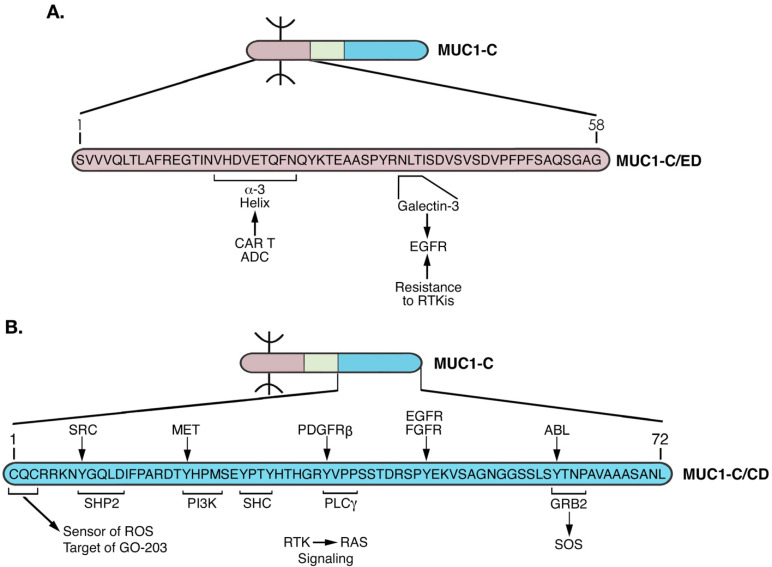
Targeting the MUC1-C extracellular and cytoplasmic domains for disruption of auto-inductive nodes and elimination of CSCs. (**A**). Antibody 3D1 generated against the MUC1-C/ED alpha-3 helix has been developed for (i) allogeneic CAR T cells that are under clinical evaluation, and (ii) ADCs that are being advanced with IND-enabling studies by the NCI NExT Program. MUC1-C forms complexes with EGFR at the cell membrane that are mediated by galectin-3 [44]. In this way, MUC1-C contributes to EGFR activation and resistance to EGFR inhibitors [101]. Antibodies generated against the alpha-4 helix are being developed to block the MUC1-C/ED interaction with galectin-3 and thereby inhibit constitutive MUC1-C-driven RTK activation. (**B**). The MUC1-C/CD CQC motif is necessary for MUC1-C homodimerization and function as an oncoprotein. Targeting the MUC1-C CQCRRKN region with the GO-203 inhibitor blocks interactions with TCF4 [61], TAK1 [69] and JAK1 [82]. GO-203 treatment also inhibits the interactions of MUC1-C with STAT3 [82] and NF-κB [77]. As a result, targeting the MUC1-C CQC motif disrupts auto-induction of MUC1-C NODES 1–3. Ongoing work is addressing another MUC1-C node that may be of importance for RTK→RAS signaling in cancer. In this regard, MUC1-C forms complexes with effectors of the RTK→RAS pathway that include PI3K [102], SHC, PLCγ and GRB2/SOS [103].

The accounting of MUC1-C functions in cancer cells supports a potential model in which MUC1-C evolved for mammalian barrier tissues to rapidly adjust to insults by mounting robust responses necessary for wound healing. Activation of these proliferative, inflammatory and repair signaling pathways in stem cells could be sustained by MUC1-C auto-inductive nodes, which we contend, if prolonged, contribute to the CSC state. In support of this model, emerging evidence has documented dependence of CSCs on MUC1-C for self-renewal capacity and tumorigenicity [65,92,94,96]. Therefore, targeting MUC1-C to directly disrupt these self-regulating nodes may be necessary for elimination of CSCs and achieving cures. Antibodies directed against the MUC1-C/ED are under development as (i) allogeneic CAR T cells that are presently under evaluation in patients with MUC1-C expressing cancers, and (ii) antibody-drug conjugates (ADCs) with support from the NCI NExT Program for IND-enabling studies (Figure 9A). These agents have the potential for targeting cancer cells that express MUC1-C on the cell surface.

Targeting MUC1-C in the nucleus will conceivably be necessary for disrupting MUC1-C-driven auto-inductive proliferative and inflammatory nodes. MUC1-C localizes to chromatin in activating (i) WNT/β-catenin, (ii) NF-κB, and (iii) STAT3 target genes that encode effectors of epigenetic reprogramming [14]. Involvement of MUC1-C in chromatin remodeling has uncovered additional nuclear functions [15,68]. An Achilles’ heel of MUC1-C is a CQC motif in the CD that is necessary for nuclear import (Figure 9B) [48]. Cell penetrating peptides targeting the MUC1-C CQC motif have been developed that are effective against CSCs (Figure 9B) [65,92,94,96]. In addition, anti-sense oligonucleotides (ASOs) have been developed to target MUC1-C addiction of CSCs [96]. PROTACs represent another approach for targeting the (i) MUC1-C/ED at the galectin-3 binding region, and/or (ii) MUC1-C/CD at directed binding motifs. The intrinsically disordered MUC1-C/CD has presented a challenge to date for the generation of small molecule inhibitors; however, ongoing research is addressing this obstacle for unstructured proteins that function as nodes in cancer progression.

## 8. Conclusions

We propose that MUC1-C-associated cancers are a consequence of the highly prevalent increases in chronic inflammation from changes in our dietary, environmental and lifestyle exposures to biotic and abiotic insults [9,14,104]. Along these lines, MUC1-C could also be a contributing factor to the marked increases in early-onset cancers [105]. Consistent with these proposals, MUC1-C contributes to the progression of adenocarcinomas, squamous cell carcinomas and certain hematologic malignancies that largely develop after reproductive age. Therefore, most MUC1-C-associated cancers have not been subject to negative selection pressures that should be occurring with emergence of the *MUC1* gene in mammals and chronic inflammation in humans.
